# Design of a Morphing Skin with Shape Memory Alloy Based on Equivalent Thermal Stress Approach

**DOI:** 10.3390/mi13060939

**Published:** 2022-06-13

**Authors:** Wei Zhang, Yueyin Ma, Xinyu Gao, Wanhua Chen, Xutao Nie

**Affiliations:** 1 College of Aerospace Science and Engineering, National University of Defense Technology, Changsha 410073, China; zhangwei8@cardc.cn (W.Z.); mayueyin@cardc.cn (Y.M.); 2 China Aerodynamics Research and Development Center, Mianyang 621000, China; gaoxinyu@cardc.cn (X.G.); chenwanhua@cardc.cn (W.C.)

**Keywords:** morphing skin, shape memory alloy, equivalent thermal strain approach, smart materials, twinned martensite

## Abstract

Shape memory alloy (SMA) is one of the potential driving devices for morphing aircraft due to its advantages of pseudoelasticity, superelasticity, and shape memory effect. Precise and fast analysis of SMA has simultaneously become a key requirement for industrial applications. In this study, a user-defined material subroutine (UMAT) was implemented and successfully applied in a three-dimensional numerical simulation in ABAQUS based on the extended Boyd–Lagoudas model. In addition to the conventional detwinned martensite (*M*_d_) and austenite (*A*), twinned martensite (*M*_t_) was also considered to model the practical transformation accurately. Then, the equivalent thermal strain approach was adopted to simplify the simulation complexity with UMAT. By resetting the thermal expansion coefficient, the thermal strain equivalent to the original phase transformation strain was generated. The approach was validated in two cases, showing consistent results with the extended Boyd–Lagoudas model and reduction in time consumption by 89.1%. Lastly, an active morphing skin integrating the single-range SMA and a stainless-steel plate was designed to realize two-way morphing. The calculated arc height variation of the skin was 3.74 mm with a relative error of 1.84% compared to the experimental result of 3.81 mm. The coupled use of UMAT and the equivalent thermal stress approach helped to reduce the challenge in modeling SMA.

## 1. Introduction

Conventional vehicles are generally designed for optimal aerodynamic efficiency in single flight status and cannot achieve the best aerodynamic efficiency across the whole flight envelope. A typical flight mission normally consists of several different processes, and combined tasks are often required. It is challenging for an ordinary fixed-wing aircraft to perform multiple tasks. Morphing foil is one of the solutions for future planes [1]. Hydraulic systems are implemented in the conventional fixed-wing plane to drive the pneumatic control surface to achieve flight control while bringing excessive mass due to complicated equipment [2]. The morphing technology based on intelligent systems has become a potential means to develop a more efficient wing [3].

The morphing wing’s in-plane stiffness needs to be low to obtain large deformation and simultaneously decrease the driving force. Furthermore, high out-of-plane stiffness is required to bear and transmit sufficient aerodynamic load. Different solutions have been investigated, such as rubber-based flexible deforming skins relying on elastic deformation, shape memory polymer (SMP) skins, morphing skins relying on rigid deformation of multiple sections, and corrugated deformation skins based on periodic instrument

Kikuta et al. established a systematic evaluation method to analyze thermoplastic polyurethanes and co-polyesters [4]. Silicone rubber was applied to variable trailing edge wings to achieve smooth and continuous deformation by Kudva et al. [5]. However, they struggled to withstand and transmit aerodynamic loads, and external continuous driving was required to maintain their deformation [6]. Corrugated skin had a better out-of-plane load-carrying capacity and could provide a large deformation in a specific direction. A variable stiffness deformable skin based on corrugated structure and SMP was developed [7,8]. One side of the ridged design was filled with SMP to guarantee a smooth aerodynamic surface. Although the aerodynamic performance of the corrugated sheet skin was comparable to some streamlined airfoils in sections with a particular Reynolds number, ensuring its smooth continuity was still a challenge [9,10,11,12].

Shape memory alloys (SMAs) have the advantages of pseudoelasticity, superelasticity, and shape memory effect, making them a favorable choice in various applications [13]. In aerodynamics, SMA is considered one of the ideal materials to drive morphing skin and foil due to its simple operation form and high energy density [14]. The weight of the aircraft can be reduced. Hence, fuel consumption can be reduced, decreasing the operating cost of the aircraft [15].

An active deforming honeycomb made of SMA was designed, achieving a maximum driving force of 300 N by heating [16]. Georges et al. [17] and Botez et al. [18] applied SMA to the skin’s up and down deflection to realize wing camber variation. Driesen et al. [19] separated the NACA0012 airfoil at the trailing edge and employed SMA wire to directly drive seven wing ribs. Ameduri et al. [20] exploited an SMA torque tube to induce a certain twist law along the blade while preserving its integrability within the structure. Kim et al. [21] designed a hybrid actuator capable of shape retention, integrated with an SMP scaffold and an SMA wire. The hybrid composite actuator could maintain a deformed state without additional current for heating by using the shape memory effects of SMP. Jodin et al. [22] provided an accurate scale flap of a civil aircraft, which could carry realistic aerodynamic loading of several tons with SMA as the macro-actuators. In Leal et al.’s study, the SMA wires were embedded into an elastic matrix and wound through each pin in turn. The restoring force by the heated SMA wires wound together pulled the two rows of pins closer to each other, thus completing the wing deformation [23,24]. The elastic potential energy stored in the passive wing bending could re-stretch the shrunken SMA filaments back to their original shape when heating stopped. Msomi et al. [25] designed an intelligent aileron using a NiTi SMA plate for the leading edge and an aluminum plate for the trailing edge. Heating the SMA plate could produce a maximum deflection of 0.57° for the smart aileron. Aso et al. [26] proposed a torsional deformation driving mechanism with two coaxial tubes. The inner one was connected to an actuator to provide torque to realize the wing’s torsional deformation. Dipalma et al. [27] explored a new autonomous morphing concept where a camber increase of 13° was realized over a spanwise section of a helicopter rotor blade with increased ambient temperature by integrating SMA on the lower surface of the blade. A conceptual hybrid design with surfaces embedded with SMA and trailing macro fiber composites was proposed to optimize the efficiency throughout the flight period [28].

SMA properties were often optimized on the basis of experimental results, which were vital for dynamic analysis [29]. Different theoretical models and algorithms were used for one-dimensional analysis [30,31,32]. Wang et al. [33,34] presented a finite element analysis (FEA) of the training behavior of a SMA wave spring actuator using a thermomechanically coupled and finite-strain SMA model. Roh et al. [35] developed an incremental formulation to predict the thermomechanical responses of the SMA strip. Oehler et al. [36] came up with a suite of optimization algorithms coupled with ABAQUS and a custom SMA constitutive model to assess morphing structure designs in an automated fashion. In Ameduri’s research [37], a descriptive finite element model underwent an optimization process performed using a proprietary code to increase the airfoil trailing edge curvature. Saputo et al. [38] used a UMAT including two phases in the ABAQUS standard finite element code environment to consider both the shape memory effect and the pseudo-elastic behavior of SMA springs.

The studies above mainly focused on experimental investigations. Although some numerical models can be found, most of them mainly involved one-dimensional simulations and considered only two phases. Moreover, it is challenging to simultaneously realize the quick and precise analysis of SMA. In this study, a UMAT was compiled and implemented in ABAQUS to model the practical three-dimensional transformation based on the extended Boyd–Lagoudas model [39,40], which is dedicated to not only the martensitic transformation between austenite and detwinned martensite but also considers twinned martensite in SMA. Then, the equivalent thermal approach [41,42] was adopted and validated in two cases using the Boyd–Lagoudas model to simplify the simulation procedure and improve efficiency. Lastly, an active morphing skin design integrating the single-range SMA and a stainless-steel plate is proposed using the equivalent thermal strain approach to realize two-way morphing.

## 2. The Extended Boyd–Lagoudas Model

The extended Boyd–Lagoudas model incorporates three-phase variations. First, the theoretical model is presented; then, a UMAT is compiled in ABAQUS and validated by experiments.

### 2.1. Mathematical Model

**Figure 1:**
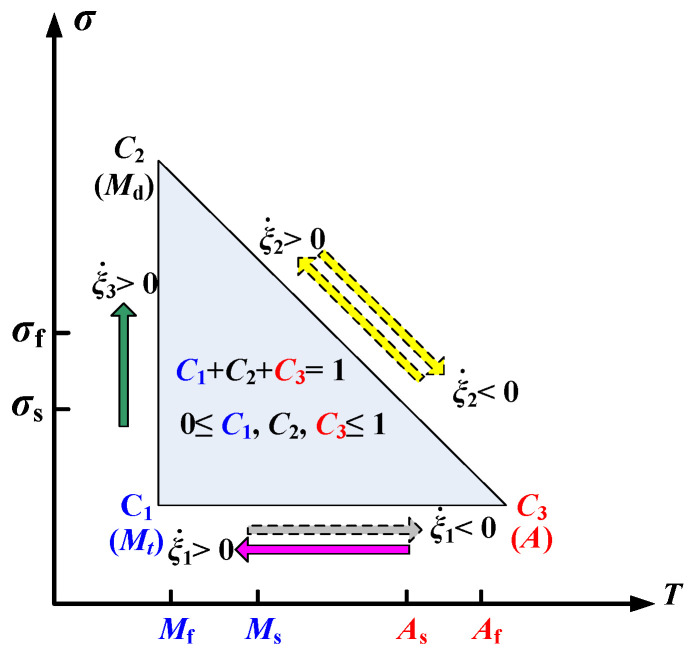
Schematic of phase transformation among three types.

In most constitutive models, the analysis of hyperelastic and shape memory effects only considers the transition between detwinned martensite and austenite. However, the actual experimental SMA behavior is more complicated. The transitions between twinned martensite and austenite and between twinned martensite and detwinned martensite also exist, as shown in Figure 1.

For instance, it is necessary to study the detwinning process of twinned martensite before SMA recovers its shape due to heating and unloading the external force. The volume fraction of each martensite should be initialized to calculate the inverse phase transformation. Therefore, three SMA phases may coexist on some loading paths meeting the following constraints:

(1) $$c_{1}+c_{2}+c_{3}=1,\;0\leq c_{1},c_{2},c_{3}\leq 1,$$

where *c*_1_, *c*_2_, and *c*_3_ represent the volume fractions of twinned martensite, detwinned martensite, and austenite, respectively.

Lagoudas and Boyd [39,40] proposed macroscopic free energy functions for the SMA to constrain constitutive behavior. Then, the phase interaction yield surface was defined to derive the constitutive relationship model and describe the phase transformation behavior. The SMA Gibbs free energy *G* includes linear thermoelastic and nonlinear phase change strengthening properties, as expressed below.

(2) $$G\left(\sigma ,T,\xi ,\varepsilon^{\mathrm{in}}\right)=-\frac{\sigma :S\left(c_{1}+c_{2}\right):\sigma }{2\rho }-\frac{1}{\rho }\sigma :\left[\alpha \left(c_{1}+c_{2}\right)\left(T-T_{0}\right)+\varepsilon^{\mathrm{in}}\right],\;+c\left(c_{1}+c_{2}\right)\left[\left(T-T_{0}\right)-T\ln\left(\frac{T}{T_{0}}\right)\right]+\left(c_{1}+c_{2}\right)\left(u_{0}-s_{0}T\right)+G^{\mathrm{mix}},$$

where ***σ***, *ε*^in^, *ξ*, *T*, and *T*_0_ are the true stress tensor, phase change strain tensor, volume fraction transformation vector, temperature, and reference temperature, respectively. ***S***, ***α***, *ρ*, *c*, *s*_0_, and *u*_0_ are the equivalent flexibility tensor, equivalent thermal expansion coefficient, density, equivalent specific heat, equivalent specific entropy, and equivalent internal energy at the reference temperature, respectively. *G*_mix_ is the mixing free energy, characterizing the mutual transformation behavior between the martensite and parent phases. The above equivalent material properties can be calculated as a function of the volume fraction of martensite and properties of every single phase. The inelastic strain *ε*^in^ exists in two processes: detwinned martensite versus austenite and twinned martensite versus detwinned martensite. It can be decomposed as follows:

(3) $$\varepsilon^{\mathrm{in}}=\varepsilon^{t}+\varepsilon^{d},$$

(4) $${\dot{\varepsilon }}^{t}=\Lambda^{t}{\dot{\xi }}_{2},$$

(5) $${\dot{\varepsilon }}^{d}=\Lambda^{d}{\dot{\xi }}_{3},$$

where *ε*^t^ is the stress-induced strain during transformation from austenite to detwinned martensite, and *ε*^d^ is the inelastic strain generated during detwinning. ***Λ***^t^ is the phase transition vector between *M*^d^ and *A*, while ***Λ***^d^ is the detwinning tensor of the change from *M*^t^ to *M*^d^, and their expressions are shown in Equations (6) and (7).

(6) $$\Lambda^{t}=\{\begin{array}{c} \frac{3}{2}H^{t}\frac{\sigma^{\prime }}{{\bar{\sigma }}^{\prime }}\left(\dot{\xi_{2}}>0\right)\\ H^{t}\frac{\varepsilon^{t-r}}{{\bar{\varepsilon }}^{t-r}}\left({\dot{\xi }}_{2}<0\right) \end{array},$$

(7) $$\Lambda^{d}=\frac{3}{2}H^{d}\frac{\sigma^{\prime }}{{\bar{\sigma }}^{\prime }}\left(\dot{\xi_{3}}>0\right),$$

where *H*^t^ and *H*^d^ correspond to the uniaxial maximum phase strain of the two types of phase transformation processes, $\sigma^{\prime }$ is the diviatoric stress tensor, ${\bar{\sigma }}^{\prime }$ is the equivalent force, *ε*^t−r^ is the phase strain vector in the martensitic inverse phase transformation process, and the sign ˙ indicates the material time derivative operation. ${\bar{\varepsilon }}^{t-r}$ is the equivalent phase strain, as defined below.

(8) $$\sigma^{\prime }=\sigma -\frac{1}{3}\mathrm{tr}\left(\sigma \right)1,$$

(9) $${\bar{\sigma }}^{\prime }=\sqrt{\frac{3}{2}{\left\|\sigma^{\prime }\right\|}^{2}},$$

(10) $${\bar{\varepsilon }}^{t-r}=\sqrt{\frac{2}{3}\left\|\varepsilon^{t-r}\right\|{}^{2}},$$

where the operator || ||^2^ represents the inner product of the vector. The thermomechanical process must satisfy the second law of thermodynamics.

(11) $$-\rho \dot{G}-\dot{\sigma }:\varepsilon -\rho s\dot{T}\geq 0,$$

where the sign: represents the double dot product operation. Furthermore, the energy transformation conditions and directions followed by the thermodynamic behavior of SMA are defined in Equation (12).

(12) $$\left(-\rho \frac{\partial G}{\partial \xi }\right)\cdot \dot{\xi }=\pi \dot{\xi }\geq 0,$$

where ***ξ*** = (*ξ*_1_, *ξ*_2_, *ξ*_3_), and *π* is the thermodynamic force vector conjugating to *ξ*. Substituting the Gibbs free energy expression into the above equation, Equations (13)–(15) are obtained whenever ${\dot{\xi }}_{1}\neq 0$, ${\dot{\xi }}_{2}\neq 0$, and ${\dot{\xi }}_{3}>0$.

(13) $$\pi_{1}=\tilde{\pi }\left(\sigma ,T\right)-f_{1}\left(\xi ,\mathrm{sgn}\left({\dot{\xi }}_{1}\right)\right).$$

(14) $$\pi_{2}=\sigma :\Lambda^{t}+\tilde{\pi }\left(\sigma ,T\right)-f_{2}\left(\xi ,\mathrm{sgn}\left({\dot{\xi }}_{2}\right)\right).$$

(15) $$\pi_{3}=-\rho \frac{\partial G}{\partial \xi_{3}}=\sigma :\Lambda^{d}-f_{3}\left(\xi \right).$$

(16) $$\tilde{\pi }\left(\sigma ,T\right)=0.5\sigma :\Delta S:\sigma +\Delta \alpha :\sigma \left(T-T_{0}\right)-\rho \Delta c\left[T-T_{0}-T\ln\left(\frac{T}{T_{0}}\right)\right]+\rho \Delta s_{0}T-\rho \Delta u_{0}.$$

In the above equations, *f*_i_ is the hardening function and depends on *c*_i_. *ξ*_i_ denotes the volume fraction conversion variations. The phase transition functions *Φ*_i_ for each type of transition process are defined according to the thermal driving force *π*_i_, as expressed in Equations (17)–(21).

(17) $$\dot{\xi_{1}}>0,\;\Phi_{1}^{+}\left(\sigma ,T,\xi \right)=\pi_{1}-Y_{1}^{+},$$

(18) $$\dot{\xi_{1}}<0,\;\Phi_{1}^{-}\left(\sigma ,T,\xi \right)=-\pi_{1}-Y_{1}^{-},$$

(19) $$\dot{\xi_{2}}>0,\;\Phi_{2}^{+}\left(\sigma ,T,\xi \right)=\pi_{2}-Y_{2}^{+},$$

(20) $$\dot{\xi_{2}}<0,\;\Phi_{2}^{-}\left(\sigma ,T,\xi \right)=-\pi_{2}-Y_{2}^{-},$$

(21) $$\dot{\xi_{3}}>0,\;\Phi_{3}\left(\sigma ,T,\xi \right)=\pi_{3}-Y_{3},$$

where $Y_{1}^{\pm }$, $Y_{2}^{\pm }$, and $Y_{3}$ are the measures of internal dissipation of the respective transformations, independent of *σ*, *T*, and***ξ***.

The phase transition function *Φ*_i_ is similar to the yield function in plastic mechanics. Therefore, analogous to the maximum plastic dissipation criterion in plastic mechanics, the phase transition can be regarded as the yield deformation. The constitutive relationship constraint can be expressed in the form of a Kuhn–Tucker inequality.

(22) $${\dot{\xi }}_{i}\geq 0,\Phi_{i}\left(\sigma ,T,\xi \right)\leq 0,\;\Phi_{i}{\dot{\xi }}_{i}=0.$$

(23) $${\dot{\xi }}_{i}\leq 0,\Phi_{i}\left(\sigma ,T,\xi \right)\leq 0,\;\Phi_{i}{\dot{\xi }}_{i}=0.$$

When ${\dot{\xi }}_{i}$ = 0 and *Φ*_i_ ≤ 0, SMA is in the thermoelastic stage of the austenite phase with no phase transition. Similarly, the SMA is in the thermoelastic stage of the martensitic phase and no phase change occurs when ${\dot{\xi }}_{i}$ = 0 and *Φ*_i_ < 0. With the phase transition function increasing to above zero, SMA begins to undergo a reverse martensitic transformation.

### 2.2. UMAT Validation

The martensite volume fraction variation was solved using the continuum tangent moduli tensors and the stresses updating algorithm of return mapping. Then, the transformation strain, equivalent stress, and tangent stiffness modulus were corrected. Afterward, a UMAT was compiled for the numerical simulation of the mechanical behavior and successfully implemented in ABAQUS using the above mathematical model to conduct the numerical simulation. The SMA used in the FEA and experimental studies was nickel–titanium (TiNi), containing Ni (49.4 wt.%) and Ti (50.6 wt.%).

**Figure 2:**
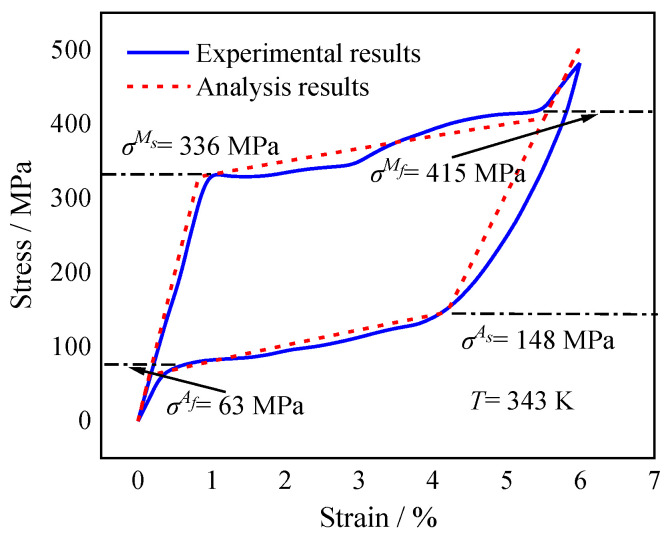
The calculated and experimental stress–strain correlations of SMA wire.

The calculated correlation between stress and strain and the experimental results [43] at 343 K are shown in Figure 2 and Table 1. The calculated stress–strain correlations are consistent with the experimental results, indicating the UMAT is appropriate for the below investigations and exhibiting the mechanical characteristics of pseudoelasticity of SMA wire. Furthermore, the four critical transformation stresses are obtained from the curves. At 343 K, the values of $\sigma^{M_{s}}$,$\;\sigma^{M_{f}}$,$\;\sigma^{A_{s}}$, and $\sigma^{A_{f}}$ were 336 MPa, 415 MPa, 148 MPa, and 63 MPa, respectively.

## 3. Equivalent Thermal Strain Approach

Compared with most SMA constitutive models, the Boyd–Lagoudas model considers the interconversion among three phases. However, it is inconvenient when applied to the three-dimensional simulation of active morphing skins. First, to ensure the accuracy of the UMAT, the model in ABAQUS solver needs to set a tiny increment size to capture the volume fraction change during the phase transformation. Thus, the accurate numerical modeling of the SMA requires an enormous time. Secondly, it is essential to define the volume fraction distribution of detwinned and twinned martensite when establishing the initial conditions for simulation. It would be tedious to perform separate martensite detwinning simulations for SMA plates and then assemble them with elastic stainless-steel plates.

The phase transition strain is gradually transformed into elastic strain by unloading the external force and raising the temperature higher than *A*_s_. The elastic stress formed overcomes the boundary constraint and restores the material’s original morphology fully or partially. Thus, the phase transition strain change is dominated by temperature, similar to thermal deformation. Then, the equivalent thermal strain approach is further adopted by resetting the thermal expansion coefficient with the same temperature-changing amplitude to produce thermal stress comparable to the initial phase change strain. The method was validated using the extended Boyd–Lagoudas constitutive relationship model in two cases.

### 3.1. Validation Case 1

**Figure 3:**
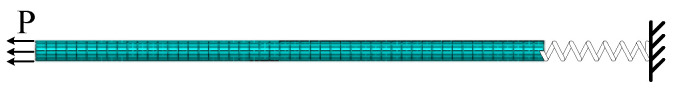
Three-dimensional mesh of SMA wire.

A three-dimensional model of SMA wire is shown in Figure 3, which had a diameter of 4 mm and a length of 100 mm. The FEA model was a 3D 20-node solid element C3D20R. The right end of the wire was connected to a spring, whose stiffness was 1000 N/mm. The stress values for the start of martensitic transformation (*σ*_s_) and completion of martensitic transformation (*σ*_f_) were about 100 MPa and 200 MPa, respectively. The other properties are presented in Table 1. The Young’s elastic modulus and Poisson’s ratio of stainless steel were *E*_steel_ = 2.1 × 10^5^ MPa and *μ*_steel_ = 0.3, respectively.

In step 1, the initial volume fractions of twinned martensite (*c*_10_), detwinned martensite (*c*_20_), and austenite (*c*_30_) were 1, 0, and 0, respectively. Both ends of the spring and the right end of the SMA wire were fixed at the temperature of 300 K. An axial stress of 140 MPa was gradually applied to the wire’s left end, resulting in partial martensitic detwinning. The volume fractions were *c*_1_ = 0.6, *c*_2_ = 0.4, and *c*_3_ = 0, consistent with the theoretical result, when *σ*_s_ and *σ*_f_ were 100 MPa and 200 MPa, respectively.

In step 2, the axial force at the left end was gradually decreased to zero. The elastic strain dropped to zero, and the phase strain and volume fraction remained unchanged.

In step 3, the left end of the spring and right end of the SMA wire were set free. Then, the SMA wire was heated until reaching 380 K, higher than *A*_f_. The wire was gradually transformed into the austenite phase, and the phase strain gradually became elastic strain. Elastic stress was formed, which pulled the spring to the left to elongate. The volume fractions were *c*_1_ = 0, *c*_2_ = 0, and *c*_3_ = 1.

In step 4, the SMA wire was cooled and gradually dropped to 280 K, below Mf. The material was progressively transformed into the twinned martensitic and detwinned martensitic phases. Consequently, the SMA wire stiffness decreased, leading the spring to shrink to the right end.

#### 3.1.1. Results of the Extended Boyd–Lagoudas Model

**Figure 4:**
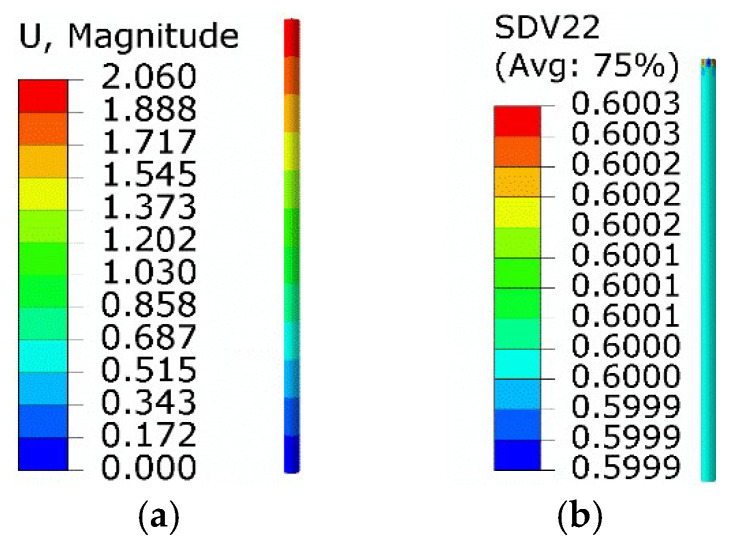
Results of step 1: (**a**) axial displacement; (**b**) the volume fraction of *M*_t_.

Corresponding numerical results were obtained using the implemented UMAT subroutine. The results of step 1 are shown in Figure 4. The equivalent stress was 140 MPa, and the maximum axial displacement was 2.06 mm. The volume fractions of twinned martensite and detwinned martensite were about 0.6 and 0.4, respectively, consistent with the theoretical predictions.

The volume fraction of twinned martensite and detwinned martensite remained unchanged in step 2. The maximum equivalent force was about 2.85 × 10^−2^ MPa, suggesting that the system had no elastic strain. The maximum axial displacement was about 1.36 mm, and the corresponding phase strain was about 1.36 × 10^−2^.

**Figure 5:**
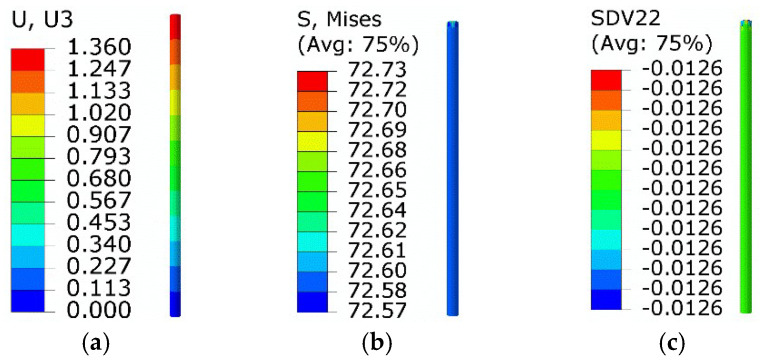
Results of step 3: (**a**) axial displacement; (**b**) equivalent stress; (**c**) volume fraction of *M*_t_.

The results of step 3 are shown in Figure 5. The twinned martensite, detwinned martensite, and austenite volume fractions were about −1.26%, 1.36%, and 99.9%, respectively. The equivalent force was about 72.7 MPa, while the axial displacement of the right end was about 0.91 mm.

**Figure 6:**
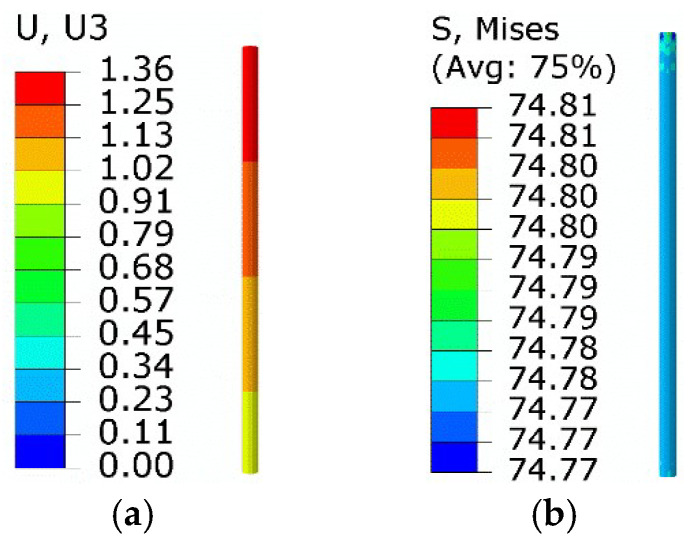
Results of step 4: (**a**) axial displacement; (**b**) equivalent stress.

The results of step 4 are shown in Figure 6. The volume fractions of twinned martensite, detwinned martensite, and austenite were about 98.77%, 1.35%, and −0.13%, respectively. The equivalent stress of SMA wire was about 74.8 MPa, and the axial displacement of the right end was about 0.94 mm. As the equivalent force was lower than the starting detwinned stress (*σ*_s_ = 100 MPa), the twinned martensite transformed from austenite did not undergo detwinning, and no phase strain was generated.

#### 3.1.2. Results of the Equivalent Thermal Strain Approach

**Figure 7:**
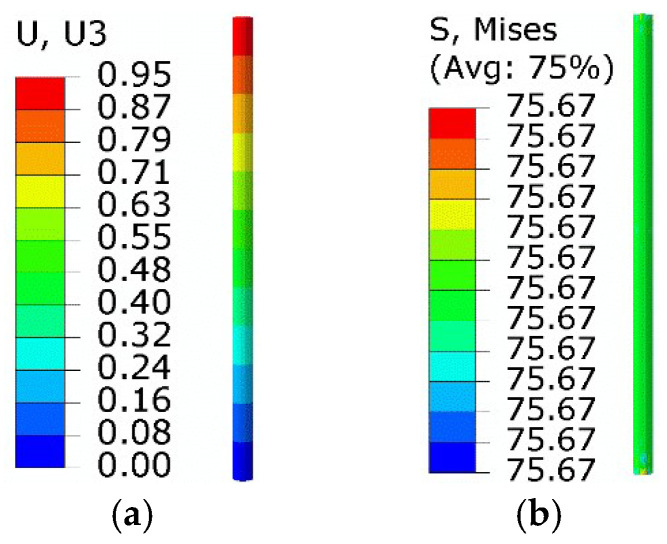
Results of step 3: (**a**) axial displacement; (**b**) equivalent stress.

The martensite inverse-phase change process in step 3 was achieved on the basis of the equivalent thermal strain approach. The thermal strain could be considered as 1.36 × 10^−2^ as the phase transition strain varied from 1.36 × 10^−2^ to 0. The original thermal expansion coefficient was 2.2 × 10^−5^ /K, and the temperature variation was set to 100 K. Thus, the new thermal expansion coefficient was redefined as −1.14 × 10^−4^ /K. The calculated results are displayed in Figure 7. Compared with the extended Boyd–Lagoudas model, the equivalent force and minimum normal displacement were 75.7 MPa and 0.95 mm, with relative errors of 4.1% and 4.4%, respectively.

**Figure 8:**
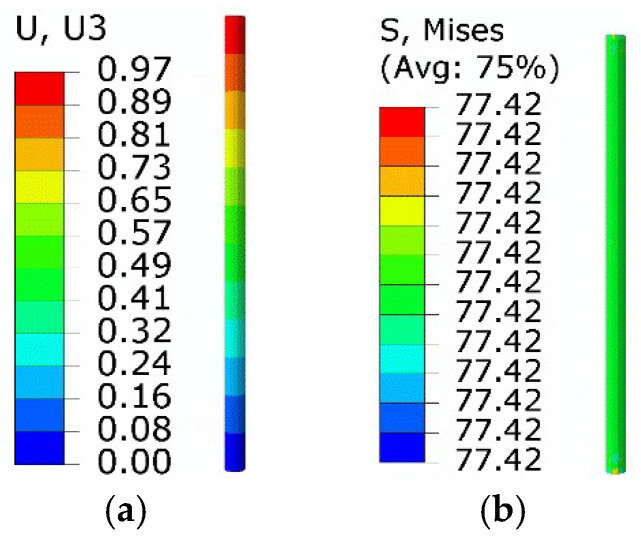
Results of step 4: (**a**) axial displacement; (**b**) equivalent stress.

The results of the martensitic positive phase transformation process in step 4 are given in Figure 8. The initial phase strain was 1.36 × 10^−2^, meaning that the thermal strain was 1.36 × 10^−2^. At the end of step 4, the SMA wire temperature recovered to the initial temperature of 280 K. The original thermal expansion coefficient was not required in this step. In addition, the detwinning of SMA wire at low temperature was studied using an elastoplastic model with an elastic modulus of 2 × 10^4^ MPa, yield stress of 100 MPa, and plastic strain of 0.034 at a stress of 200 MPa. Compared with the extended Boyd–Lagoudas model, the equivalent force and normal displacement of the right end were 77.4 MPa and 0.973 mm, with relative errors of 3.48% and 3.51%, respectively.

### 3.2. Validation Case 2

**Figure 9:**
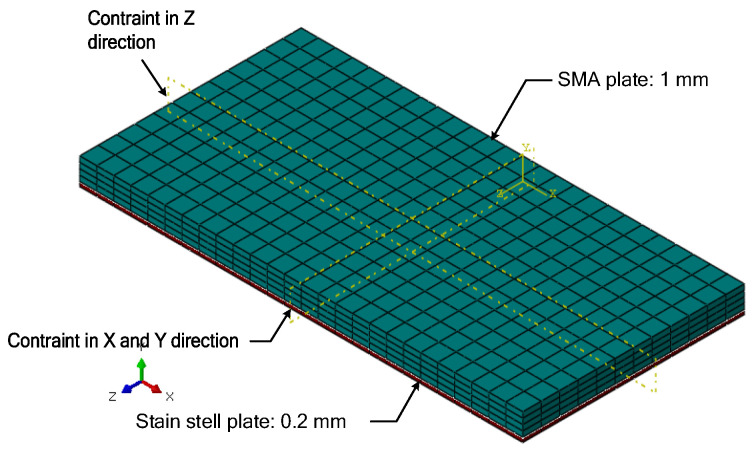
Three-dimensional structure mesh of SMA.

The composite plate shown in Figure 9 comprised a 1 mm thick SMA plate and a 0.2 mm thick metal plate. Their length and width were 20 mm and 10 mm, respectively. The C3D20R unit was implemented for the CAE grid division. The SMA material properties were as follows: *E*_A_ = 4 × 10^4^ MPa, *E*_M_ = 2 × 10^4^ MPa, *μ*_M_ = *μ*_A_ = 0.33, *M*_s_ = 245 K, *M*_f_ = 230 K, *A*_s_ = 270 K, *A*_f_ = 280 K, and *H* = 0.034. The thermal expansion coefficient of austenitic and martensite was considered zero in the initialization. The properties of stainless steel were *E*_steel_ = 2.1 × 10^5^ MPa and *μ*_steel_ = 0.3.

#### 3.2.1. Results of the Extended Boyd–Lagoudas Model

**Figure 10:**
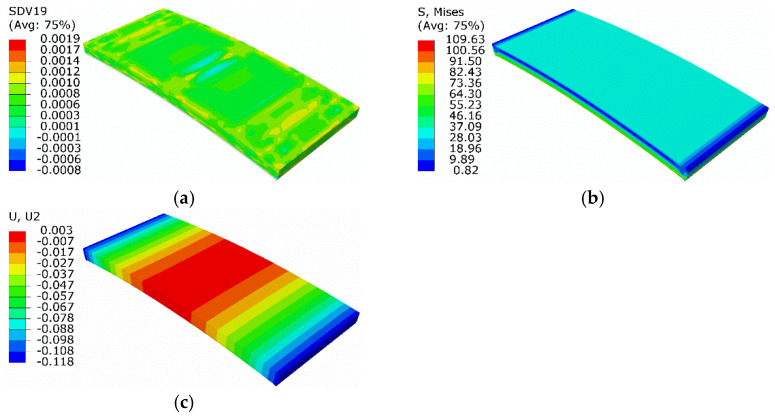
Results at the end of temperature rising: (**a**) volume fraction of detwinned martensite; (**b**) equivalent stress; (**c**) normal displacement.

The initial temperature of the whole plate was 220 K. The SMA plate was in a pure detwinned martensitic state with a phase transformation strain of −0.034, i.e., under a compressive state. Then, the SMA became austenitic when its temperature gradually exceeded the inverse-phase transition temperature by heating. Correspondingly, the phase transformation strain progressively transitioned into the elastic strain, forming elastic stress and forcing the metal plate to bend. As the temperature was below *A*_s_, only the transformation of detwinned martensite to austenite was considered. Moreover, the twinned martensite was not involved in this case. Results at the end of temperature rising are shown in Figure 10. The volume fraction of detwinned martensite was about 5.5 × 10^−4^, which is close to zero and can be regarded as a complete transition to austenite. The maximum equivalent force was about 109.6 MPa, and the minimum normal displacement was about −0.118 mm.

#### 3.2.2. Results of the Equivalent Thermal Strain Approach

**Figure 11:**
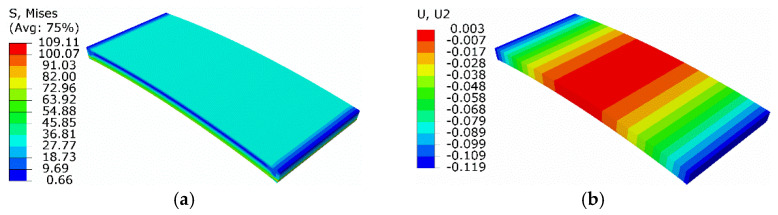
Results of simulation based on equivalent thermal strain approach: (**a**) equivalent stress; (**b**) normal displacement.

On the basis of the equivalent thermal strain approach, the above process was studied numerically. The initial phase strain was −0.034 before turning to zero, suggesting that the equated thermal strain was 0.034. The temperature increased to 300 K, above *A*_f_. Thus, the new thermal expansion coefficient was redefined as 4.25 × 10^−4^ /K. Results are shown in Figure 11.

Compared with the extended Boyd–Lagoudas model, the maximum equivalent force and minimum normal displacement were 109.1 MPa and −0.119 mm, with relative errors of 0.46% and 0.85%, respectively.

According to the comparison above, the equivalent thermal strain approach results were consistent with the Boyd–Lagoudas model, including the stress and normal displacement. Using the same hardware and grid size in case 2, the time cost of the equivalent thermal strain approach was 184 s while the Boyd–Lagoudas model took 1682 s, representing a reduction of 89.1%. Moreover, compared with the Boyd–Lagoudas model, the equivalent thermal strain approach could equate the twinned martensite distribution by setting the temperature boundary conditions with a straightforward operation. Therefore, the equivalent thermal strain technique could be applied to the efficient and accurate numerical simulation of the SMA mechanism.

## 4. Design of Active Morphing Skin

**Figure 12:**
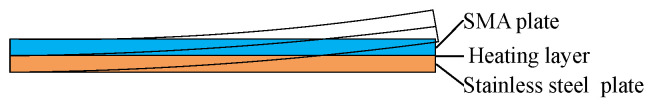
Schematic of active morphing skin.

An active morphing skin integrating a one-way SMA with a stainless-steel plate is proposed in Figure 12. A flexible stainless-steel plate was implemented as the driving instrument. After the one-way SMA completes its actuation, it is cooled to the low-temperature martensite state. The elastic potential energy stored in the stainless-steel plate forces SMA to recover its original state, thus realizing the repeated deformation drive of SMA. The design avoids the drawbacks of two-way SMA, which is restricted by its minor phase change recovery force and poor driving capacity.

**Figure 13:**
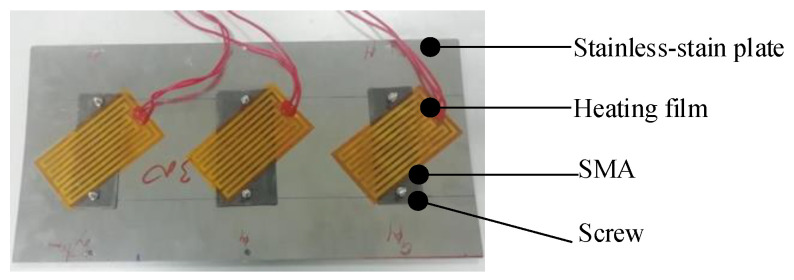
Active morphing skin prototype.

A specimen of the active morphing skin is shown in Figure 13. The elastic matrix was a stainless-steel plate, whose thickness, length, and width were 0.5 mm, 300 mm, and 150 mm, respectively. Three SMA plates were attached, all with a thickness of 1 mm and a length of 80 mm. Their widths were 30 mm, 40 mm and 30 mm, respectively, from left to right. The SMA properties were as follows: *E*_A_ = 5.6 × 10^4^ MPa, *E*_M_ = 2.5 × 10^4^ MPa, *μ*_A_ = *μ*_M_ = 0.33, *α*_A_ = *α*_M_ = 2.2 × 10^−5^ /K, *M*_s_ = 295 K, *M*_f_ = 255 K, *A*_s_ = 323 K, *A*_f_ = 368 K, *H* = 0.034, *σ*_s_ = 100 MPa, and *σ*_f_ = 200 MPa. The properties of stainless steel were *E*_steel_ = 2.1 × 10^5^ MPa and *μ*_steel_ = 0.3. The SMA plate was heated by a Zhenglong film with a heating power of 30 W, while the cooling approach was natural convection by air.

Before being connected by screws, the SMA plates were trained according to the following steps: first, the SMA plate was clamped in the mold into an electric furnace at 800 °C for 40 min of training. Then, the mold was discharged from the furnace, and the SMA plate was flattened after cooling to room temperature. Finally, it was placed it into the furnace at 200 °C for 5 min to recover its original state.

The above steps were repeated 50 times. Under the room-temperature condition, the SMA plate was bent with a radius of 87.5 mm. Then, the SMA plate was pressed to induce partial detwinning. The SMA plate recovered to be approximately flat when the pressure load was removed.

### 4.1. Simulation of SMA Plate Flattening

**Figure 14:**
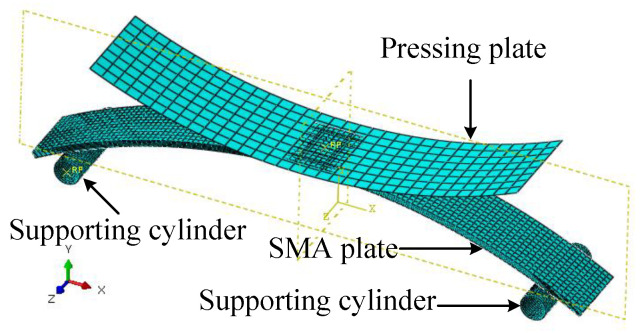
Numerical model of flattening.

The simulation of the flattening process was carried out to achieve the characteristic of martensite detwinning. The numerical model shown in Figure 14 included an SMA plate, a pressing plate, and two support cylinders. The SMA plate was defined as a three-dimensional elastomer. The constitutive relationship was determined by the equivalent plastic model with a yield stress of 100 MPa and a plastic strain of 0.034 at the equivalent stress of 200 MPa.

Both support cylinders were fixed and restrained to support the left and right ends of the SMA plate. The simulation process was divided into two steps. First, the pressing plate was moved downward in the negative *Y*-axis direction for 15 mm to compress the SMA plate. Second, the pressing plate was moved upward to its initial position.

**Figure 15:**
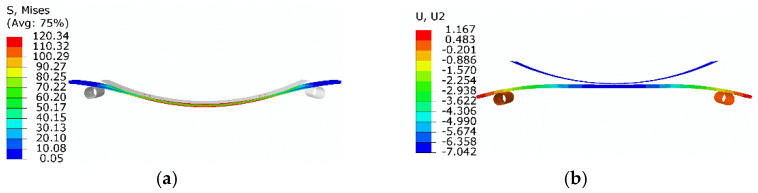
Numerical results of flattening: (**a**) results of the first step; (**b**) results when the pressing plate is segregated.

Figure 15a gives the equivalent stress distribution of the first step. The SMA plate middle area changed from convex to concave under the pressing. The maximum equivalent stress was about 120.3 MPa, and the corresponding plastic strain was 6.9 × 10^−3^. The thickness of the region where the equivalent stress above *σ*_s_ (100 MPa) was about 0.25 mm indicates that the plastic deformation thickness was about 0.25 mm.

Figure 15b shows the displacement when the pressing plate was segregated from the SMA plate. The middle of the plastically deformed SMA plate recovered to nearly flat. To press the SMA plate flat, the maximum plastic strain of the SMA plate was about 6.9 × 10^−3^. The thickness of the plastic deformation zone was about 0.25 mm, which was used in the subsequent modeling.

### 4.2. Modeling of SMA Plate Driving

On the basis of the equivalent thermal strain approach mechanism, simulations under high temperature and low temperature were conducted to explore the skin’s actual morphing process.

Under high-temperature conditions, the equivalent thermal strain was calculated according to the phase change strain and actual thermal stress generated by heating. Then, the corresponding temperature boundary conditions and variations were calculated by the actual thermal expansion coefficient. Under low-temperature conditions, the equivalent thermal strain was determined by the phase transformation strain without considering the actual thermal strain. The corresponding equivalent thermal expansion coefficient was achieved by the temperature variation.

#### 4.2.1. Transformation into Austenite by Heating

**Figure 16:**
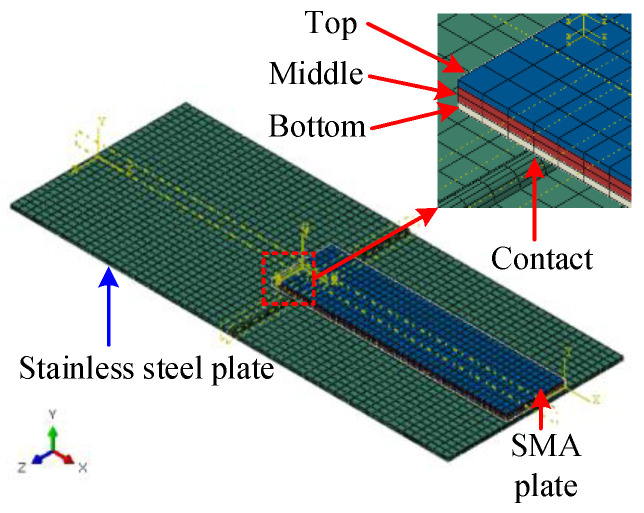
Semi-symmetric model of the active morphing skin.

A semi-symmetric model is shown in Figure 16, including a 0.5 mm thick stainless-steel plate and a 1 mm thick SMA plate. The length and width of the stainless-steel plate were 75 mm and 30 mm. The length and width of the SMA plate were 40 mm and 10 mm, respectively. The SMA plate numerical model was divided into three layers along the thickness direction to describe the detwinning distribution of twinned martensite. According to the thickness of the plastic deformation zone in Figure 15, the top, middle, and bottom layer thicknesses were 0.25 mm, 0.5 mm, and 0.25 mm, respectively. A semicircular lug boss was implemented on the top surface of the stainless-steel plate, and it was in contact with the lower surface of the bottom layer of the SMA plate to model the connection to transmit the bending force.

**Figure 17:**
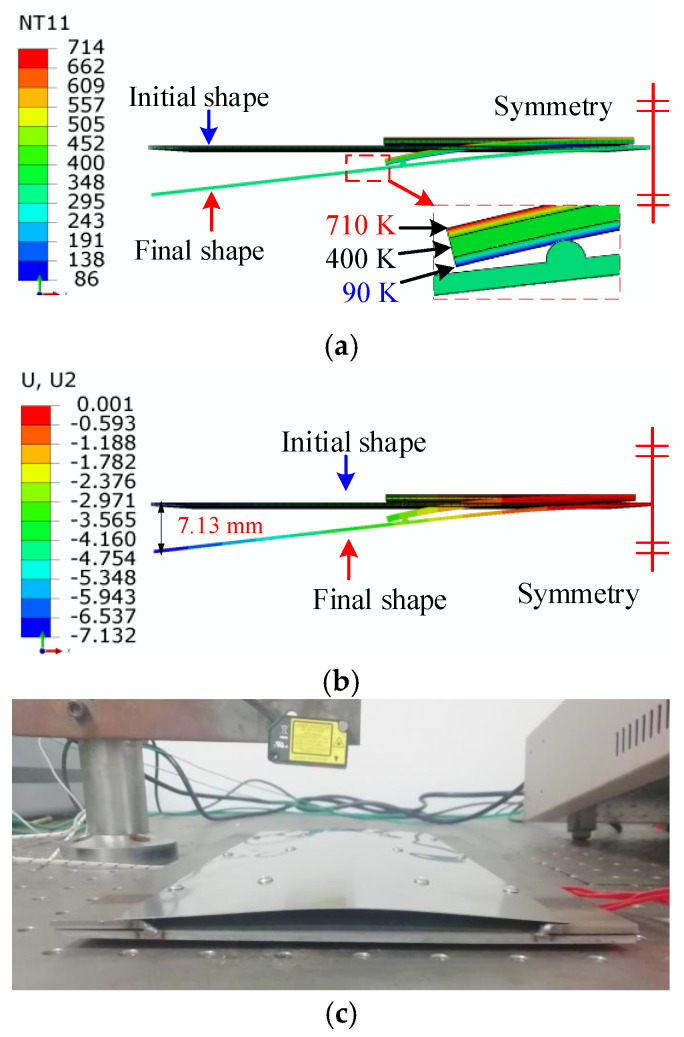
The deformation driven by SMA plate under high-temperature conditions: (**a**) temperature distribution; (**b**) displacement in the *Y*-direction; (**c**) experimental measurement of deformation.

The initial temperature was 300 K, and the corresponding thermal expansion coefficient of SMA was 2.2 × 10^−5^ /K. Different temperatures were initialized to model each SMA layer’s phase change strain and thermal strain, as shown in Figure 17a. The upper surface temperature of the top SMA layer was set to 710 K. The upper and lower surface temperatures of the middle layer were set to 400 K. The lower surface temperature on the bottom layer was set to 90 K.

The top layer’s phase transformation strain was 6.9 × 10^−3^ from Figure 15, and the thermal strain was 2.2 × 10^−3^, i.e., the product of thermal expansion coefficient and the temperature difference. Therefore, the equivalent thermal strain of the top SMA layer was about 9.1 × 10^−3^, i.e., the sum of the phase transformation strain and the thermal strain. The equivalent thermal strain of the bottom SMA layer was about 4.7 × 10^−3^, i.e., the difference between the phase change strain and the thermal strain. The middle layer of the SMA plate was twinned martensite, which had no phase change strain and did not require thermal strain equivalence. The numerical results are presented in Figure 17b, showing that the left end of the stainless-steel plate was shifted downward by 7.13 mm.

The experimental deformation was measured by the laser displacement sensors in Figure 17c. The measured arc height of the stainless-steel plate was 7.86 mm, indicating that the relative error of the numerical results was about 9.3%.

#### 4.2.2. Transformation into Martensite by Cooling

The initial temperature was still 300 K with the same numerical model. The plastic model was implemented to describe the SMA constitutive relationship with the yield stress of 100 MPa. The plastic strain was 0.034 at the equivalent stress of 200 MPa. Under this condition, the temperature remained nearly unchanged. Therefore, the thermal strain was not considered, and only the effect of phase transformation strain was taken into account.

**Figure 18:**
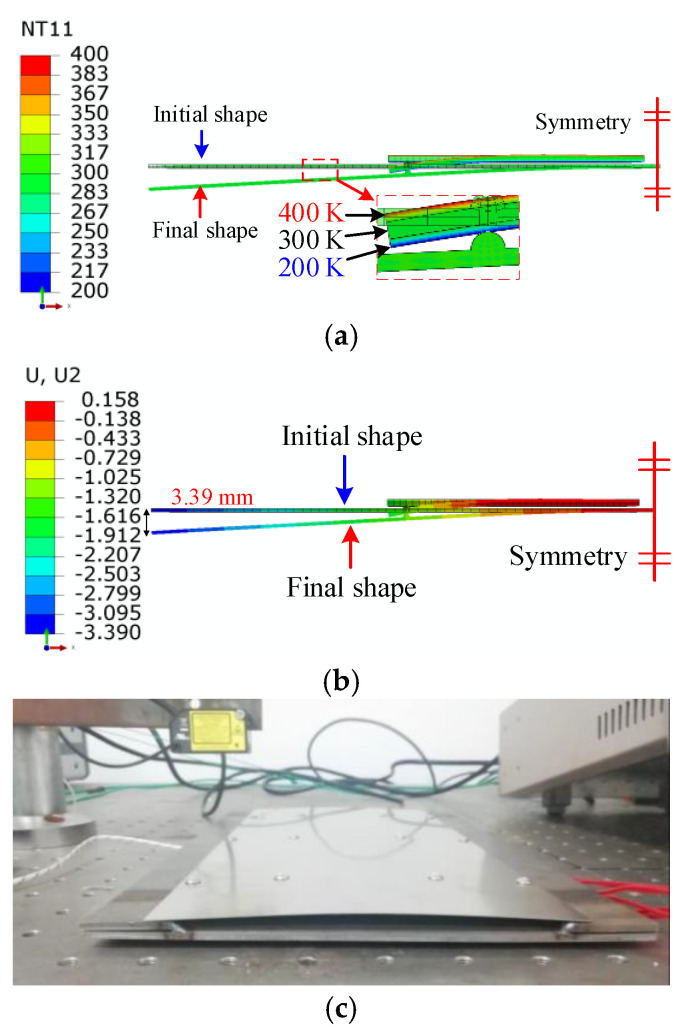
The deformation driven by SMA plate under the low-temperature conditions: (**a**) temperature distribution; (**b**) displacement in the *Y*-direction; (**c**) experimental measurement of deformation.

In Figure 18a, the top layer gradually changed from 400 K on the upper surface to 300 K on the lower surface. The whole middle layer was 300 K. The bottom layer gradually changed from 300 K on the upper surface to 200 K on the lower surface. The thermal expansion coefficients of the top and bottom SMA layers were reset to 6.9 × 10^−5^ /K, indicating an equivalent thermal strain of 6.9 × 10^−3^.

There was no transformation strain due to twinned martensite in the middle SMA layer; thus, thermal strain equivalence was not employed. The left end of the stainless-steel plate moved downward by 3.39 mm, as shown in Figure 18b. The experimental deformation is presented in Figure 18c. The arc height of the stainless-steel plate was 4.05 mm, indicating that the relative error of the nuerical results was about 16.3%.

The shape memory recovery effect was indicated and validated by FEA and the experimental study, as shown in Figure 14, Figure 15 and Figure 16. The original SMA plate was arc-shaped, as shown in Figure 14. With the training of the pressing plate, the SMA plate changed to nearly flat. The maximum deformation magnitude was 7.04 mm, as shown in Figure 15b. As shown in Figure 17, the SMA plates drove the skin to its original arc shape, and the maximum displacement in the *Y*-direction was 7.13 mm after heating. Upon cooling, the composite plate tended to recover to a flat shape due to the stored elastic potential energy in the stainless-steel plate. The alternate deformation of the skin was achieved by varying the temperature. The calculated stainless-steel plate arc height ranged from 3.39 mm to 7.13 mm, representing an amplitude of 3.74 mm. The corresponding displacement in the experimental result varied from 4.05 mm to 7.86 mm, representing an amplitude of 3.81 mm. Thus, the simulation’s relative error was about 1.84%, proving the accuracy of the equivalent thermal strain approach in simulating the application of SMA in morphing foil.

## 5. Conclusions

This study presented the numerical and experimental investigations of a morphing skin, providing a fast and precise approach for the subsequent wing finite element analysis.

(1) The complicated transformation process among three SMA phases, twinned martensite, detwinned martensite, and austenite, was fully considered on the basis of the extended Boyd–Lagoudas model. A UMAT implementing the actual interaction was achieved, and a three-dimensional numerical simulation was realized in ABAQUS, showing consistent results with the experiment.
(2) An equivalent thermal strain approach was adopted to simplify the SMA simulation procedure and reduce consumption. The approach was validated by the extended Boyd–Lagoudas model in two cases with a maximum relative error of 4.4%, and the time cost was reduced by 89.1%. The alternate use of UMAT and the simplified approach helped to reduce the challenge in subsequently modeling the wing using FEA.
(3) An active two-way morphing skin driven by SMA and stainless steel was designed. The simulated arc height variation was 3.74 mm with a relative error of 1.84%, compared to the experimental result of 3.81 mm.

## Figures and Tables

**Table 1:** Material properties for SMA wire.

Material Properties	Value
Wire diameter	1 mm
Martensite start temperature *M*_s_	302 K
Martensite finish temperature *M*_f_	291 K
Austenite start temperature *A*_s_	326 K
Austenitic finish temperature *A*_f_	336 K
Young’s martensitic modulus *E*_M_	2 × 10^4^ MPa
Young’s austenitic modulus *E*_A_	4 × 10^4^ MPa
Poisson’s ratio (equal for both phases) *μ*	0.33
Coefficient of thermal expansion for the martensite *α*_M_	2.2 × 10^−5^
Coefficient of thermal expansion for the austenite *α*_A_	2.2 × 10^−5^
Maximum transformation strain *H*	0.034
Stress influence coefficient (equal for both phases) *ρ*∆s_0_	−0.3131
$\mathrm{Stress}\;\mathrm{for}\;\mathrm{initiation}\;\mathrm{of}\;\mathrm{martensitic}\;\mathrm{transformation}\;\sigma^{M_{s}}$	336 MPa
$\mathrm{Stress}\;\mathrm{for}\;\mathrm{completion}\;\mathrm{of}\;\mathrm{martensitic}\;\mathrm{transformation}\;\sigma^{M_{f}}$	415 MPa
$\mathrm{Stress}\;\mathrm{for}\;\mathrm{initiation}\;\mathrm{of}\;\mathrm{austenitic}\;\mathrm{transformation}\;\sigma^{A_{s}}$	148 MPa
$\mathrm{Stress}\;\mathrm{for}\;\mathrm{completion}\;\mathrm{of}\;\mathrm{austenitic}\;\mathrm{transformation}\;\sigma^{A_{f}}$	63 MPa

## Data Availability

Data are contained within the article.
